# Molecular Identification and Drug Susceptibility of *Leishmania* spp. Clinical Isolates Collected from Two Regions of Oaxaca, Mexico

**DOI:** 10.3390/microorganisms13020220

**Published:** 2025-01-21

**Authors:** Adriana Moreno-Rodríguez, Ada Sarai Martin del Campo-Colín, Luis Roberto Domínguez-Díaz, Ana Livia Posadas-Jiménez, Félix Matadamas-Martínez, Lilián Yépez-Mulia

**Affiliations:** 1 Laboratorio de Estudios Epidemiológicos, Clínicos, Diseños Experimentales e Investigación, Facultad de Ciencias Químicas, Universidad Autónoma “Benito Juárez” de Oaxaca, Avenida Universidad S/N, Ex Hacienda Cinco Señores, Oaxaca 68120, Mexico; arimor10@hotmail.com; 2 Unidad de Investigación Médica en Enfermedades Infecciosas y Parasitarias, UMAE Hospital de Pediatría, Centro Médico Nacional Siglo XXI, Instituto Mexicano del Seguro Social, Mexico City 06720, Mexico; ada.martinc@gmail.com; 3 Centro de Patología Clínica, Licenciatura en Ciencias Biomédicas, Universidad de la Sierra Sur, UNSIS, Guillermo Rojas s/n esq. Av. Universidad, Col. Universitaria, Miahuatlán de Porfirio Díaz, Oaxaca 70800, Mexico; rober_0403@hotmail.com; 4 Programa de Prevención y Control de la Enfermedad de Chagas, Departamento de Prevención y Control de Enfermedades Transmitidas por Vector de la Dirección de Prevención y Promoción de la Salud, Servicios de Salud de Oaxaca, Oaxaca 68000, Mexico; pojilivia@gmail.com

**Keywords:** skin smears, ITS1 PCR, drug susceptibility

## Abstract

Pentavalent antimonials are the first line for leishmaniasis treatment, although they induce many adverse side effects and treatment failure and parasite resistance have been detected. Cutaneous leishmaniasis is the main clinical manifestation of the disease in Oaxaca State, Mexico; however, its presence is under-registered, and information about the *Leishmania* species that circulate and cause the disease in the region is limited. In this study, the presence of *Leishmania* was analyzed in 24 skin smears and 2 biopsies from lesions suspicious for leishmaniasis in inhabitants of the Tehuantepec Isthmus and Papaloapan Basin regions, Oaxaca State. By ITS1-PCR, the species of clinical isolates were identified. Moreover, the susceptibility of clinical isolates to leishmanicidal drugs was assessed. Skin smears were negative for the presence of *Leishmania* spp.; meanwhile, parasite amastigotes were observed in tissue biopsies; however, by ITS1-PCR, 46% of the samples were determined to be positive for the parasite. Six clinical isolates were identified as *L. mexicana* and had lower susceptibility to Miltefosine and Amphotericin B than the *L. mexicana* reference strain. No leishmanicidal activity of Glucantime was detected. Further studies with increased patient sample sizes and genotypic studies will describe in detail parasite susceptibility to reference drugs in the region.

## 1. Introduction

Protozoan species of the genus *Leishmania* are the causative agents of leishmaniasis in endemic areas of Northeastern Africa, Southern Europe, the Middle East, Mexico, and Central and South America. Leishmaniasis is included by the World Health Organization (WHO) as a tropical neglected disease. It is transmitted to humans, domestic, and wild animals by sandfly bite, mainly those of the *Lutzomyia* (New World) and *Phlebotomus* (Old World) genus. The WHO estimates that 700,000 to 1 million new cases are registered annually and approximately 350 million people are at risk of leishmaniasis [1]. Leishmaniasis is usually more common in rural than in urban areas; the main risks are seen among gum tree harvesters and cocoa and banana farmers; and it is linked to deforestation, irrigation schemes, and urbanization, with climate and environmental changes impacting the vector populations and their distribution [2,3].

There are three clinical manifestations of leishmaniasis: cutaneous (CL), mucocutaneous (MCL), and visceral (VL).

CL lesions are characterized by the presence of one or more ulcerated skin lesions with raised edges and a localized or diffuse granulomatous background; MCL leads to the partial or total destruction of mucous membranes of the nose, mouth, and throat, and VL is the most serious and is almost always fatal if untreated [4]. CL lesions may be confused with other skin conditions, such as staphylococcal or streptococcal infections, mycobacterial cutaneous lesions, leprosy, fungal infection, cancer, and sarcoidosis [5]. The clinical presentation of CL lesions may vary depending on host immunity and the causative *Leishmania* species [6]. The identification of *Leishmania* spp. by molecular approaches is relevant since different species of *Leishmania* may coexist and cause lesions of very similar appearance; therefore, its clinical diagnosis is difficult. However, it has been reported that *L. mexicana* causes one or more ulcerated skin lesions localized or sometimes diffuse with raised edges and a granulomatous background. On the other hand, *L. amazonensis* causes ulcerated localized CL, frequently causes diffuse and disseminated CL, and can develop into MCL [7].

The Pan American Health Organization (PAHO) reported 252,988 total cases of CL and MCL in America, with an annual average of 42,166 cases from 2017 to 2022. CL and MCL cases were reduced everywhere except for Mexico, Guatemala, and Panama. In Mexico, an increase in CL cases was registered from 2018 (576 cases) to 2019 (1014 cases), and 1281 cases were reported in 2022 [6].

Leishmaniasis treatment depends on different factors such as the type of disease, parasite species, concomitant pathologies, and geographical location. Pentavalent antimonials (SbVs), sodium stibogluconate (Pentostam), and meglumine antimoniate (Glucantime) were first used at the beginning of the 20th century; however, they are considered the first-line treatments against most forms of leishmaniasis, and they have also been used as a reference to compare the efficacy of other potential treatments. Amphotericin B, pentamidine isethionate, and Miltefosine constitute the other therapeutic drugs used to treat leishmaniasis [5,6]. Glucantime and Pentostam have several limitations including high costs, the difficult route of administration (parenteral), administration for 20–30 days, and many adverse side effects such as cardiotoxicity, increased liver function tests, urea, creatinine, anorexia, nausea, vomiting, myalgia, and arthralgia. Intralesional infiltration has been proposed as an alternative treatment route to reduce adverse effects [8,9,10]. In Mexico, SbV therapy has been recommended to treat CL; however, in 2022, a cure rate of 60% was registered [6].

Treatment failure can be due to host factors, including parasite characteristics such as parasite species and drug concentration, among others [11,12,13,14,15]. In some cases, SbVs are used in combination with second-line drugs such as Amphotericin B and Miltefosine. Importantly, treatment failure of SbVs in India, Peru, Latin America, Asia, and Africa has been reported [10,16,17,18,19,20,21,22,23,24,25]. In addition, CL diagnosis can be difficult, since different species of *Leishmania* may coexist and cause lesions of very similar appearance. In the case of coinfection with more than one species with different clinical implications, the identification of *Leishmania* species is essential to choose appropriate and early treatment against various clinical manifestations of leishmaniasis. It has been shown that the response to drugs can vary between *Leishmania* species and among strains of the same species, drug susceptibility determination is also important for disease treatment [5,11,12,13,14,15].

In this regard, PAHO recommends the use of intralesional administration of SbVs in patients with CL caused by *L. braziliensis* or *L. amazonensis*; meanwhile, Miltefosine is recommended for the treatment of CL caused by *L. panamensis*, *L. mexicana*, *L. guyanensis*, or *L. braziliensis* [5]. *Leishmania* species causative of CL are sensitive to SbVs; however, drug resistance is a serious problem for leishmaniasis treatment in some endemic areas [6,10,11,12,13,14,15,16].

The direct detection of amastigotes in smears or histopathological sections obtained from skin lesions is the gold standard for CL diagnosis, though it can also be achieved by parasite in vitro cultivation [26]. Although these techniques are specific, they are not sensitive enough. In this regard, molecular methods have shown promise for parasite diagnosis and species identification. Among the molecular techniques used, polymerase chain reaction (PCR) is the most successful technique for leishmaniasis diagnosis and species identification [27,28,29]. In this regard, the amplification of molecular targets by PCR such as minicircle kinetoplast DNA (kDNA) [30], the miniexon gene (spliced leader RNA) [31], and the internal transcribed spacer (ITS1), as well as by the gp63 [32,33] and by the restriction fragment length polymorphism of ITS1 PCR (ITS1 PCR-RFLP) [32], is amongst the most commonly used methods for the diagnosis and/or identification of *Leishmania* species. It was demonstrated that kDNA-PCR and ITS1 PCR showed 100% sensitivity for parasite diagnosis of CL samples; meanwhile, parasite microscopy and parasite culture detected 43% and 29% of the true positive samples, respectively [26]. In addition, the simultaneous diagnosis and identification of the parasite species from the Mexican endemic states of Tabasco, Campeche, and Veracruz using ITS1 PCR-RFLP was achieved [32]. *L. mexicana* caused localized CL cases in Tabasco and Campeche States; meanwhile, *L. mexicana* and *L. amazonensis* caused Diffuse CL (DCL) cases in Tabasco and Veracruz States. In addition, *L. mexicana* and *L. braziliensis* complex caused localized CL cases in Campeche State. It is known that Oaxaca State, located on the Southern Pacific coast of Mexico, harbors vectors for *Leishmania* transmission, and the presence of CL has been registered [34]; however, information about the *Leishmania* species that circulate and cause the disease in the region is limited. In addition, the detection of parasites in smears of skin lesions is employed for disease diagnosis; however, due to its low sensitivity, many false negative samples are registered, accounting for the under-registration of the disease. Our aim was to analyze, in a cross-sectional study, the presence of *Leishmania* spp. in skin lesions suspicious of leishmaniasis from inhabitants of the endemic areas of Tehuantepec Isthmus and Papaloapan Basin, Oaxaca State. The presence of *Leishmania* was searched in 24 skin smears, 2 skin biopsies, and 1 parasite culture (26 samples). By ITS1 PCR, the parasite presence was also analyzed. In addition, *Leishmania* spp. was identified by ITS1 PCR-RFLP, and the susceptibility of clinical isolates to the leishmanicidal reference drugs was evaluated.

## 2. Materials and Methods

### 2.1. Skin Samples

Sample collection was carried out as recommended in the Report of the Meeting of the WHO Expert Committee in Geneva from March 2010 for leishmaniasis control [35].

**Figure 1:**
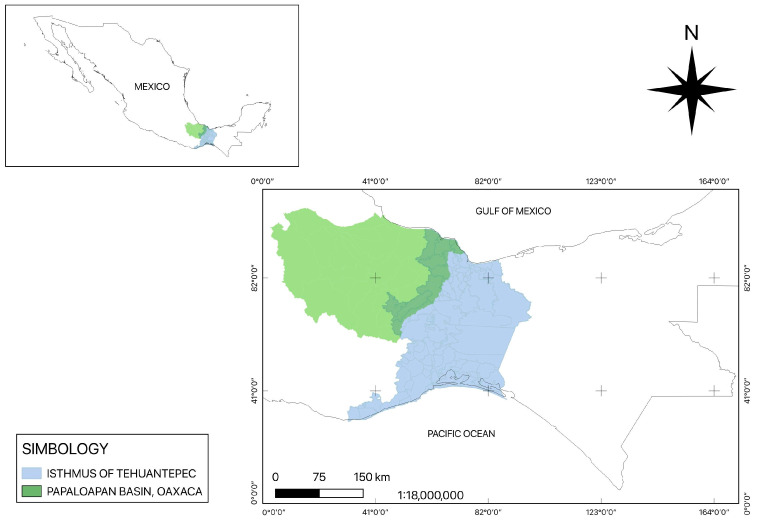
Tehuantepec Isthmus and Papaloapan Basin regions of Oaxaca State.

Skin samples with suspicious leishmaniasis lesions were collected from patients from the Isthmus of Tehuantepec and Papaloapan Basin, Oaxaca State, Mexico, from 2018 to 2024. (Figure 1). Sampled individuals had an average age of 61 ± 35 years, and 22 out of 26 samples were men and 4 were women; they were mostly farmers and continuously exposed to the vector. Their skin lesion durations ranged from months to years.

In the present cross-sectional study, 10 out of 26 patients had previously received Glucantime^®^ [Merial, Lyon, France] six had treatment failure (samples 2, 3, 5, 12, 19, and 20) and four of them were cured (8, 9,15, 18). Patients 8 and 9 were treated with antibiotics and patient 18 had antifungal treatment before Glucantime^®^ treatment. Two (6 and 13) and three (7, 16, 17) patients were treated only with antibiotics and antifungals, respectively. The other nine patients had no parasite diagnosis or treatment (1, 4, 10, 11, 14, 21–24). Two patients (25 and 26) were clinically diagnosed with DCL, but at the moment tissue samples were collected, treatment had not been administered.

### 2.2. Leishmania spp. Diagnosis in Skin Samples

For the parasitological diagnosis, imprints taken from the skin lesions were stained with Giemsa; in the case of samples 25 and 26, a biopsy was taken and stained with hematoxylin and eosin, both samples were obtained from patients clinically diagnosed with DCL. Slides were observed in a Zeiss Primostar microscope [Carl Zeiss MicroImaging GmbH, Gôttingen, Germany] with a 100× objective, and images were captured using a 48 MP camera. Image processing was performed by using Adobe Photoshop 2024 version.

In addition, skin samples were inoculated in Schneider’s Insect Medium [Sigma-Aldrich, St. Louis, MO, USA] supplemented with 10% heat-inactivated Fetal Bovine Serum (FBS) and 100 U/mL of penicillin plus 100 μg/mL of streptomycin (Sigma-Aldrich). Skin samples were incubated at 26° C for 7 days to allow the transformation of viable amastigotes into promastigotes. The promastigotes were subcultured in Schneider’s medium several times until their axenization was achieved. The clinical isolates were used to identify the *Leishmania* species.

### 2.3. Detection of Leishmania spp. in Tissue Samples by ITS1 PCR

A portion of skin tissue scraped with cytobrush and/or aspirated from the lesion was maintained in ethanol and used for molecular diagnosis. Parasite detection was achieved by ITS1 PCR, as previously recommended [32]. For this, DNA from skin samples was obtained by phenol–chloroform extraction, and the parasite DNA that encodes for internal transcribed spacers 1 (ITS1) was amplified by PCR using the LITSR (5′CTG GAT CAT TTTCCG ATG 3′) and L5.8S (5′ TGA TAC CAC TTA TCG CACT T 3′) primers. When tissue samples were too small, the ITS1 sequence was amplified directly from the sample using the TerraTM PCR Direct Polymerase Mix Kit (Takara Clontech, San Jose, CA, USA), according to the supplier’s recommendations. DNA from the reference strain *L. mexicana* MNYC/BZ/62/M379 (obtained from Dr. Nogueda-Torres) was included as a positive control. The expected amplicons (300–350 bp) were electrophoretically run in 1% agarose gel. Images were captured with a photodocumenter (UVITEC, Cambridge, UK).

### 2.4. Identification of Leishmania Species

DNA was extracted from axenic clinical isolates, and the *Leishmania* spp was identified by ITS1 PCR-RFLP, as previously recommended [32], with some modifications. For this, the ITS1 sequence was amplified as described before and restricted with the *Hae*III enzyme. DNA from the reference strains *Leishmania mexicana* MNYC/BZ/62/M379 and *L. amazonensis* MHOM/BR/73/M2269 (ATCC 50131) was also included. Restriction fragments were electrophoretically separated in 8% polyacrylamide gel. Images were captured with a photodocumenter (UVITEC, Cambridge, UK).

### 2.5. Reference Drugs

Amphotericin B and Miltefosine were purchased from Sigma-Aldrich. The pharmaceutical formulation of Glucantime^®^, containing 81 mg/mL of SbV, was used according to the instructions of the supplier (Merial, Lyon, France).

### 2.6. Evaluation of the Leishmanicidal Activity Against Amastigotes of Leishmania Isolates, Cytotoxicity, and the Selectivity Index of Reference Drugs

Amastigote susceptibility to reference drugs was evaluated in vitro, as previously recommended [36]. In brief, 5 × 10^4^ murine macrophages (cell line J774.2, ATCC ^®^TIB-67) and stationary promastigotes from parasite isolates, at a 1:10 ratio, were seeded in 200 μL/well of RPMI-1640 culture medium supplemented with 10% heat-inactivated FBS and 100 U/mL of penicillin plus 100 μg/mL of streptomycin (Invitro, Mexico City, Mexico) in 96-well plates and incubated for 48 h at 37 °C and 5% CO_2_ in a humidity chamber. The reference strain of *L. mexicana* was included. The cells were then washed several times with RPMI medium to remove free non-infective promastigotes, and the final washing medium was replaced with 200 μL/well of culture medium containing different concentrations of Glucantime^®^ (0.78 to 100 μg/mL), Miltefosine (at 0.78 to 100 μg/mL), and Amphotericin B (at 0.078 to 10 μg/mL). After plates were incubated for another 48 h at 37 °C in 5% CO_2_, the culture medium was replaced with an equal volume of lysis solution (RPMI-1640 and 0.01% SDS) and maintained at room temperature for 20 min. The lysis solution was then replaced with Schneider’s medium followed by incubation at 26 °C for another 4 to 5 days to allow the transformation of viable amastigotes into promastigotes and their subsequent proliferation. Then, resazurin solution (20 µL at 2.5 mM) was added to each well and the plates were incubated for 3 h at room temperature. Finally, fluorescence emission was measured in a fluorometer (Infinite 200, Tecan Group Ltd., Männedorf, Switzerland) at a 544 nm excitation and a 590 nm emission wavelength. All assays were carried out in triplicate. The half maximal inhibitory concentration (IC_50_) was determined by a Probit analysis.

The cytotoxicity of reference drugs was tested using the murine macrophage cell line J774.2 (ATCC^®^TIB-67). In brief, macrophages were seeded (5 × 10^4^ cells/well) in 96-well flat-bottomed microplates and allowed to adhere for 24 h at 37 °C in 5% CO_2_. The culture medium was then replaced with different concentrations of the drugs (as previously mentioned), followed by incubation for another 48 h. All assays were carried out in triplicate. Thereafter, resazurin solution (20 µL at 2.5 mM) was added to each well and the plates were incubated for another 3 h. The fluorescence emission was measured as described above. All assays were carried out in triplicate. The half maximal cytotoxicity concentration (CC_50_) was determined by a Probit analysis. The selectivity index (SI) values of the reference drugs were calculated as the ratio of cytotoxicity to leishmanicidal activity (SI = CC_50_/IC_50_).

## 3. Results

### 3.1. Detection of Leishmania Amastigotes in Skin Lesions

**Figure 2:**
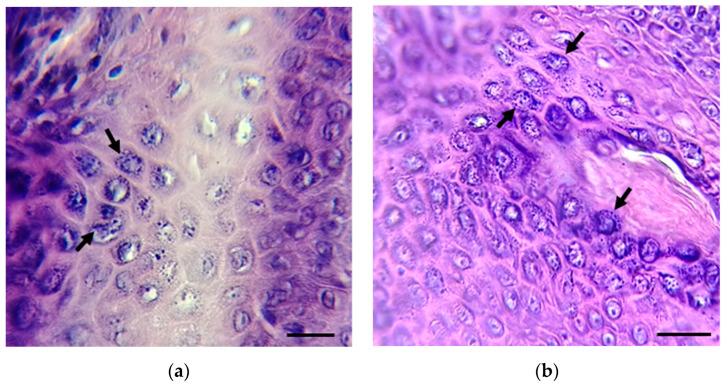
Biopsies from patients 25 (**a**) and 26 (**b**) were stained with hematoxylin and eosin. The presence of amastigotes (black arrows) was detected. Scale bar = 10 µm. Amastigote images in (**a**,**b**) are focused on the original images in Figure S1a,b.

Imprints from skin lesions (1–24) and two biopsies (25–26) from inhabitants of the Isthmus of Tehuantepec and Papaloapan basin, Oaxaca State, Mexico, with suspected leishmaniasis were analyzed for the presence of *Leishmania* spp. Most skin lesions were localized in different ear and face areas, the chest, arms, and legs. Skin imprints (1–24) were negative for the presence of *Leishmania* spp.; meanwhile, parasite amastigotes were observed in the two tissue biopsies (25 and 26) (Figure 2).

### 3.2. Molecular Detection of Leishmania spp. in Tissue Samples

**Figure 3:**
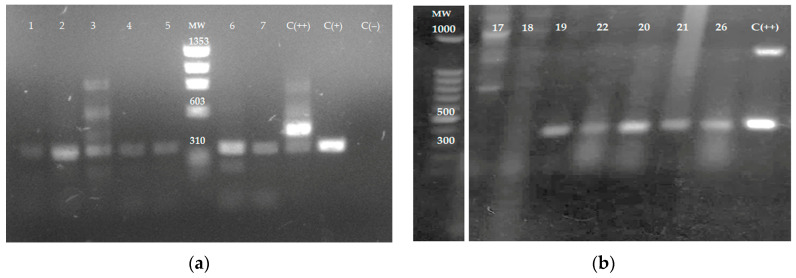
The amplification of ITS1 sequence by PCR using DNA obtained from tissue samples. DNA from skin samples was obtained and the internal transcribed spacers 1 (ITS1) sequence was amplified by PCR using the LITSR and L5.8S primers. (**a**) skin samples 1 to 7; C (+)—*L. mexicana* reference strain—and C (++)—mouse leg tissue infected with *L. mexicana*, positive controls; C (−): Uninfected mouse leg tissue negative control. MW: ΦX174 DNA-*HaeIII* Digest. (**b**) Skin samples 17–22 and 26; C (++): *L. mexicana* promastigotes were added to non-infected mouse leg tissue as a positive control. MW; 100 bp DNA. The agarose gel electrophoresis image in (**b**) is cropped from the original gel image provided in Figure S2.

The ITS1 sequence was amplified by PCR using DNA obtained from tissue samples and electrophoretically separated on 1% agarose gel. Amplification products from samples 1 to 7 (Figure 3a), 19–22, and 26 (Figure 3b) were analyzed in agarose gels. In total, 12 out of 26 samples amplified by ITS1 PCR had fragments of 300–350 bp, indicating the presence of *Leishmania* spp. Samples 17 and 18 in Figure 3b represent samples negative for the presence of the parasite. Figure 3b is cropped from the original gel image (Figure S2).

### 3.3. Clinical Isolates

To allow the transformation of viable amastigotes from skin tissue into promastigotes, tissue samples were also cultured in Schneider’s medium. The differentiation to promastigote was only obtained from samples 2, 7, 19, and 22–25. However, the axenization of promastigotes from sample 2 was not achieved due to its high bacterial and fungal contamination. Parasite isolates were named MHOM/MX/2018/UABJOFCQEPS (sample 7), MHOM/MX/2018/UABJOFCQFPA (sample 19), MHOM/MX/2018/UABJOFCQPPB (sample 22), MHOM/MX/2019/UABJOFCQSJA (sample 23), MHOM/MX/2019/UABJOFCQAFR (sample 24), and MHOM/MX/2021/UABJOFCQAXES (sample 25) following the WHO nomenclature (2010).

Table S1 presents general information about results obtained in Section 3.1, Section 3.2 and Section 3.3.

### 3.4. Identification of Leishmania Species

**Figure 4:**
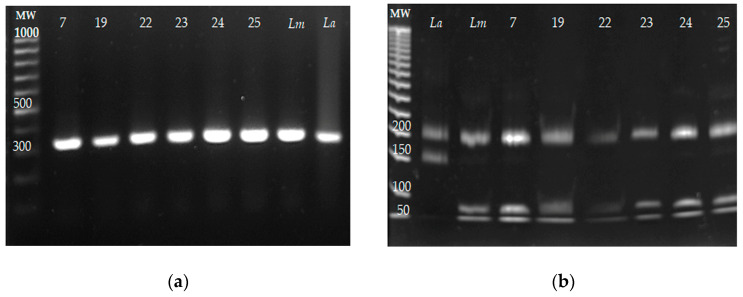
The identification of *Leishmania* species of the six parasite isolates by ITS1 PCR-RFLP. DNA from the clinical isolates was obtained, and the ITS1 sequence was amplified by PCR and restricted with the *Hae*III enzyme. DNA from the reference strains *Leishmania mexicana* MNYC/BZ/62/M379 and *L. amazonensis* MHOM/BR/73/M2269 was included as the control. (**a**) ITS1 amplification fragments from the clinical isolates *L mexicana* (*Lm*) and *L. amazonensis* (*La*). (**b**) ITS1 amplicons were restricted with the *Hae*III enzyme, and restriction fragments were electrophoretically separated on 8% polyacrylamide gel.

The identification of *Leishmania* species by ITS1 PCR-RFLP was carried out from the DNA purified from the six parasite isolates (7, 19, 22–25). The reference strains of *L. mexicana* and *L. amazonensis* were included as controls. The ITS1 PCR amplification products and their restriction products are presented in Figure 4a and Figure 4b, respectively. The sizes of the fragments obtained from the six isolates were 200, 80, and 40 bp, and the same fragment lengths were obtained for *L. mexicana*; meanwhile, fragments of 220 and 140 bp were obtained for the reference strain of *L. amazonensis*. These results indicated that the six isolates belonged to *L. mexicana*, and no co-infection with other *Leishmania* species was detected.

### 3.5. Susceptibility of Parasite Isolates to Leishmanicidal Drugs

The susceptibility of the amastigote stage of the clinical isolates to the leishmanicidal drugs Amphotericin B, Miltefosine, and Glucantime^®^ was evaluated, following the methodology previously recommended [37]. The reference strain *L. mexicana* was included as a control. The IC_50_ values obtained by Probit analysis are presented in Table 1. It was observed that the six isolates were less susceptible to Amphotericin B and Miltefosine. The IC_50_ value for the pharmaceutical presentation of Glucantime^®^ was determined to be >273 µM. Regarding Amphotericin B, isolates were 2.9 to 7.7 times less susceptible than the reference strain; isolates 22 and 25 showed the highest IC_50_ values. For Miltefosine, isolates were 1.78 to 4 times less susceptible to the drug than the reference strain. Isolates 22 and 24 had the highest IC_50_ values for Miltefosine. The reference drugs had SIs against the six isolates lower than the *L. mexicana* reference strain. 

## 4. Discussion

Pentavalent antimonials are still the primary drugs used for the treatment of different forms of leishmaniasis, although *Leishmania* species differ in their susceptibility to SbVs. Therefore, the identification of *Leishmania* species is important since the clinical manifestations of CL will depend on the parasite spp. and the host immune response [10,11,12,13,14,37]. Moreover, *Leishmania* species identification and parasite drug susceptibility are relevant for CL treatment, particularly if more than one species is found in the same region [11,12,13,14,15].

In Mexico, CL is diagnosed by the direct detection of amastigotes in skin smears, and there is a poor record of leishmaniasis cases in Oaxaca State, Mexico. Regarding treatment, CL is treated with SbVs; however, in 2022, PAHO registered a cure rate of 60% among patients treated [6].

In the present cross-sectional study, 24 skin imprints were negative for the presence of *Leishmania* spp.; meanwhile, parasite amastigotes were observed in two tissue biopsies (samples 25 and 26). The low sensitivity of the direct detection of the parasite has been proposed to be due to the presence of few amastigotes in lesions [38]; in addition, most of the skin samples collected had chronic lesions, and it is known that CL lesions can last up to 20 years, leading to scarring and deformation. In contrast, 12 out of 26 samples (46%) were determined as positive by PCR amplification of the ITS1 sequence. It has been well documented that ITS1 PCR is more sensitive than direct observation of the parasite based on skin smears or parasite culture [26]. Four samples of the twelve PCR-positive samples did not have a parasitological diagnosis or received no treatment; one sample was clinically diagnosed with DCL but without any treatment, while another five had been previously treated with Glucantime^®^ and two more had received antifungal or antibiotic treatment. ITS1 PCR results confirmed its higher sensitivity than the direct methods allowing the parasite’s detection in tissue samples with Glucantime^®^ treatment failure and patients previously misdiagnosed. Moreover, in samples without parasite diagnosis, *Leishmania* presence was detected. The health authorities were notified about PCR-positive patients and will take care of their adequate treatment.

Six clinical isolates were obtained from the 26 skin samples, and *L. mexicana* was identified as the causative species of leishmaniasis, including in sample 25, which had been clinically diagnosed as DCL. No coinfection with other *Leishmania* species was detected. Furthermore, it was important to determine the susceptibility of isolates to Glucantime^®^, Amphotericin B, and Miltefosine, since these reference drugs are recommended for CL treatment.

When the susceptibility of the six isolates to the reference drugs was evaluated, it was shown that both Miltefosine and Amphotericin B had lower leishmanicidal activity against all isolates in comparison to the reference strain. Regarding the anti-*Leishmania* activity of the SbV pharmaceutical formulation, no activity was detected even at 273 µM. Isolates 22 and 25 had the highest IC_50_ values for Amphotericin B and isolates 22 and 24 had the highest IC_50_ values for Miltefosine. It is worth mentioning that individuals 22 and 24 had not yet been diagnosed and treated before the samples were collected. Patient 25 received no previous treatment but was clinically diagnosed with DCL. Isolate 19 was less susceptible to Amphotericin B and Miltefosine, and the patient had been previously treated with Glucantime^®^ and had treatment failure. Interestingly, patient 7 had been previously treated with antifungal drugs, and the isolate had higher IC_50_ values for Amphotericin B and Miltefosine than the reference strain. Moreover, the reference drugs had SIs lower against the six isolates than the *L. mexicana* reference strain. These values indicated their lower selectivity against the parasite isolates, indicating their lower leishmanicidal activity.

It is well known that the adverse side effects of Glucantime^®^ induce patients to stop the treatment and administration of an inadequate dose of SbVs, and the misdiagnosis of the lesions followed by the administration of antifungals may account for treatment failure and the induction of parasite resistance to anti-*Leishmania* drugs.

This cross-sectional study included 26 patients; further studies with increased patient sample size and genotypic studies will allow us to describe in more detail the parasite susceptibility to reference drugs in the region.

It may be necessary to try combinatory therapy with anti-*Leishmania* drugs that have synergistic or additive activity with different targets that could reduce the therapy duration and dose, reducing their toxicity and the appearance of parasite drug resistance.

## 5. Conclusions

Altogether, the data confirm the low sensitivity of parasite detection in skin smears of lesions that may account for the under-registered CL cases observed in the region. Parasite culture also had low sensitivity. ITS1 PCR detected *Leishmania* parasites in patient samples with therapy failure. Clinical isolates were obtained from six patients, and *L. mexicana* was identified as the causative agent of CL. Importantly, the parasite isolates obtained from patients without previous treatment showed lower susceptibility to the reference anti-*Leishmania* drugs. Therefore, these data light up the need to account for new, efficacious, non-toxic, and low-cost therapeutic agents.

## Figures and Tables

**Table 1:** The leishmanicidal activity (IC_50_) *, cytotoxicity (CC_50_) *, and selectivity index (SI) ** of Amphotericin B, Miltefosine, and Glucantime^®^.

*L. mexicana* Isolates	Amphotericin B IC_50_ µM ± SD	SI	Miltefosine IC_50_ µM ± SD	SI	Glucantime^®^ IC_50_ µM ± SD	SI
*L. mexicana* MNYC/BZ/62/M379	0.19 ± 0.04	46	2.11 ± 0.83	66	>273	1
MHOM/MX/2018/UABJOFCQEPS (Sample 7)	0.61 ± 0.29	14	8.17 ± 3.06	17	>273	1
MHOM/MX/2018/UABJOFCQFPA (Sample 19)	0.38 ± 0.03	23	5.77 ± 0.55	24	>273	1
MHOM/MX/2018/UABJOFCQPPB (Sample 22)	1.08 ± 0.21	8	9.32 ± 1.241	15	>273	1
MHOM/MX/2019/UABJOFCQSJA (Sample 23)	0.60 ± 0.01	15	4.50 ± 1.31	31	>273	1
MHOM/MX/2019/UABJOFCQAFR (Sample 24)	0.71 ± 0.09	12	9.07 ± 2.36	15	>273	1
MHOM/MX/2021/UABJOFCQAXES (Sample 25)	1.17 ± 0.03	8	7.13 ± 1.57	19	>273	1
Cell line J774.2 (ATCC^®^TIB-67)	8.78 ± 1.62	-	138.56 ± 13.26	-	>273	-

* The half maximal inhibitory concentration was determined by Probit analysis. ** The selectivity index (SI) values of reference drugs were calculated as the ratio of cytotoxicity to leishmanicidal activity (SI = CC_50_/IC_50_).

## Data Availability

The original contributions presented in the study are included in the article/supplementary material, further inquiries can be directed to the corresponding authors.

## Supplementary Materials

The following supporting information can be downloaded at https://www.mdpi.com/article/10.3390/microorganisms13020220/s1, Figure S1a,b: Original images of amastigotes of Figure 2a,b; Figure S2: Original gel image used to prepare Figure 3b; Table S1: General information about patients’ parasite diagnosis, treatment status, and data obtained in this study.
