# Supplementary material for: Molecular Identification and Drug Susceptibility of Leishmania spp. Clinical Isolates Collected from Two Regions of Oaxaca, Mexico
Source: Microorganisms. 2025 Jan 21;13(2):220. doi: 10.3390/microorganisms13020220 (PMC11857778; doi:10.3390/microorganisms13020220)
Supplement: Supplementary file 1 [file microorganisms-13-00220-s001.zip › Table S1.pdf]

Table S1: General information about patients´ parasite diagnosis, treatment status, and data obtained in this study.

Samples number	Lesion localization	Observations	Diagnostic Test		
			Skin smear	ITS1 PCR	Axenization
1	Right ear	Without parasite diagnosis<br>Without treatment	Negative	Positive	Negative
2	Left ear	Glucantime Treatment Failure	Negative	Positive	Negative
3	Right ear	Glucantime Treatment Failure	Negative	Positive	Negative
4	Right ear	Without parasite diagnosis<br>Without treatment	Negative	Positive	Negative
5	Right ear	Antimicrobial therapy<br>Glucantime Treatment Failure	Negative	Positive	Negative
6	Right ear	Antimicrobial therapy	Negative	Positive	Negative
7	Right ear	Fungal diagnosis<br>Antifungal therapy	Negative	Positive	Positive
8	Nasopharyngeal	Antibiotic treatment<br>Glucantime Treatment Lesion healing	Negative	Negative	Negative
9	Nose and Mouth	Antibiotic treatment<br>Glucantime Treatment Lesion healing	Negative	Negative	Negative
10	Right cheek	Without parasite diagnosis<br>Without treatment	Negative	Negative	Negative
11	Right lacrimal	Without parasite diagnosis<br>Without treatment	Negative	Negative	Negative
12	Right ear	Glucantime Treatment Failure	Negative	ND	Negative
13	Left ear	Antimicrobial therapy	Negative	Negative	Negative
14	Right ear	Without parasite diagnosis<br>Without treatment	Negative	Negative	Negative
15	Left cheekbone	Glucantime Treatment Lesion healing	Negative	Negative	Negative
16	Left arm	Antifungal therapy	Negative	Negative	Negative
17	Dorsum, back and legs	Antifungal therapy	Negative	Negative	Negative
18	Left front area	Antifungal therapy<br>Glucantime Treatment Lesion healing	Negative	Negative	Negative

19	Left ear	Glucantime Treatment Failure	Negative	Positive	Positive
20	Left ear	Glucantime Treatment Failure	Negative	Positive	Negative
21	Left ear	Without parasite diagnosis antibiotic treatment	Negative	Positive	Negative
22	Left ear	Without parasite diagnosis without treatment	Negative	Positive	Positive
23	Left ear	Without parasite diagnosis	Negative	ND	Positive
24	Left ear	Without parasite diagnosis	Negative	ND	Positive
25	Legs	Clinical diagnosis with DCL	Negative<br>Biopsy positive	Negative	Positive
26	Abdomen	Clinical diagnosis with DCL	Negative<br>Biopsy positive	Positive	Negative

ND: not determined for insufficient DNA amount; DCL: Diffused cutaneous leishmaniasis
