# Supplementary figures and images for: Molecular Identification and Drug Susceptibility of Leishmania spp. Clinical Isolates Collected from Two Regions of Oaxaca, Mexico
Source: Microorganisms. 2025 Jan 21;13(2):220. doi: 10.3390/microorganisms13020220 (PMC11857778; doi:10.3390/microorganisms13020220)

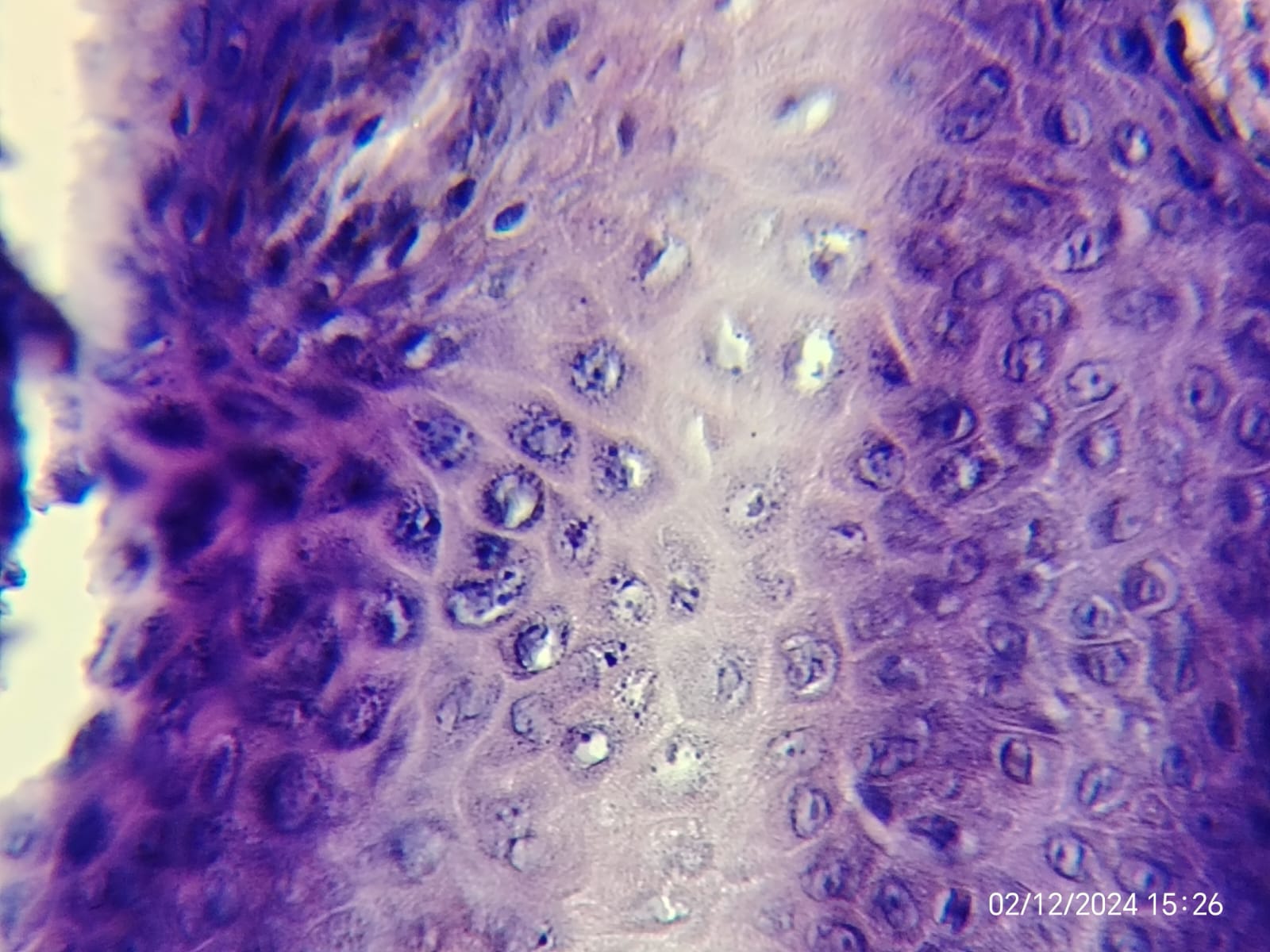

Supplement: Supplementary file 1 [file microorganisms-13-00220-s001.zip › Figure S1a.jpeg]

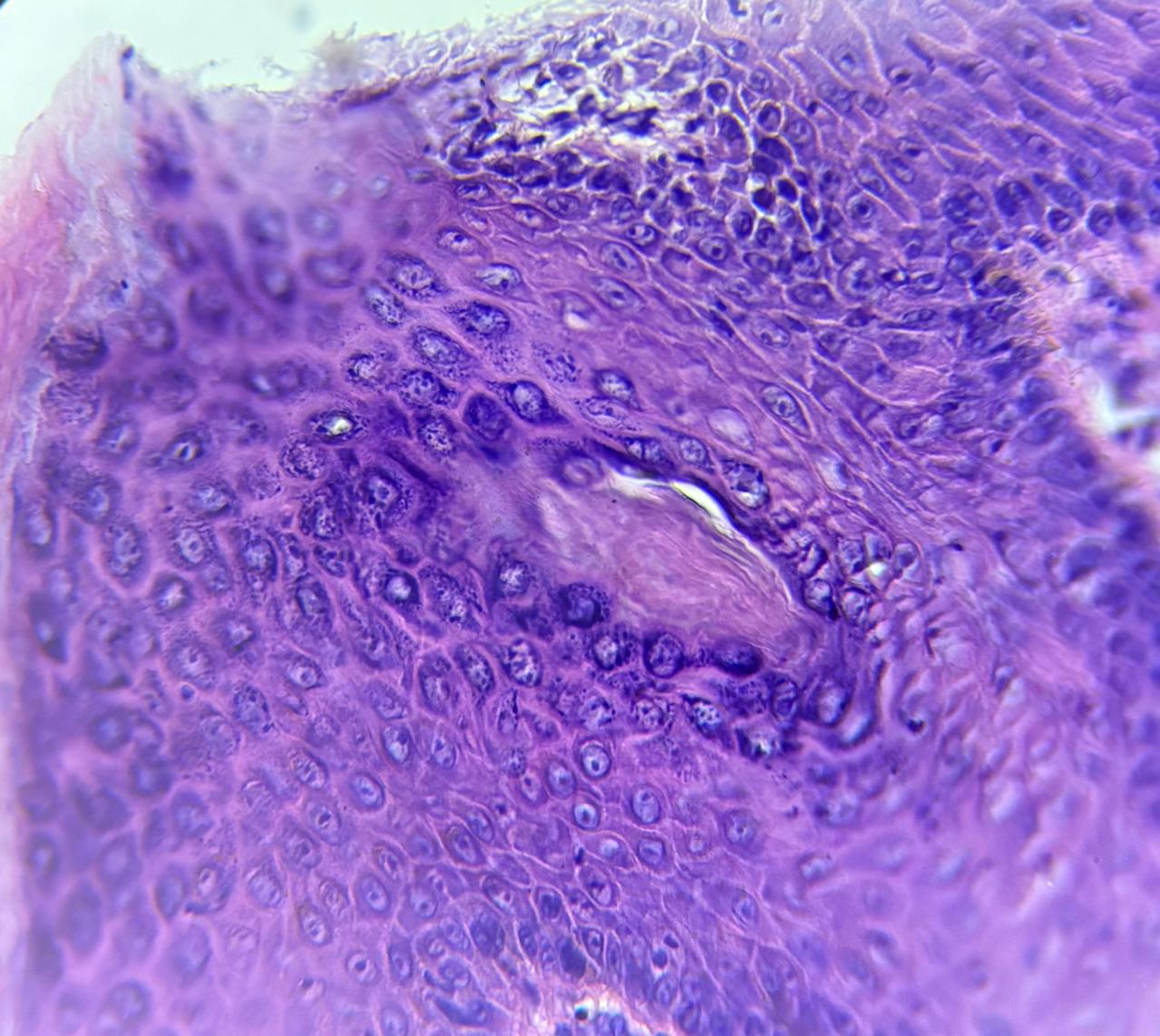

Supplement: Supplementary file 1 [file microorganisms-13-00220-s001.zip › Figure S1b.jpeg]

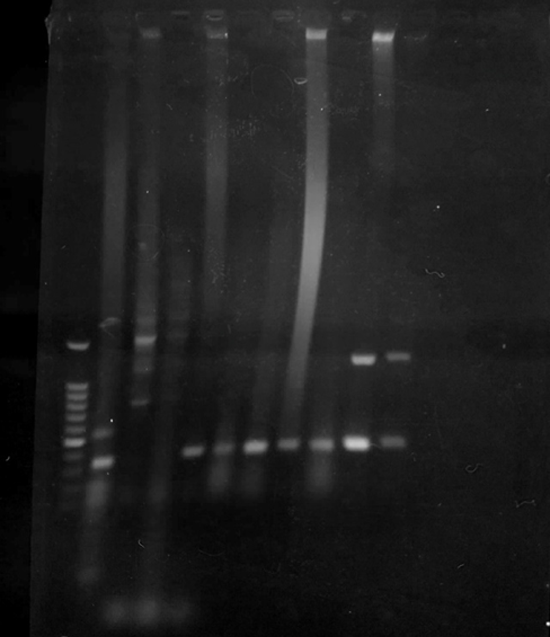

Supplement: Supplementary file 1 [file microorganisms-13-00220-s001.zip › Figure S2.tif]
